# Ag_2_S/CdS/TiO_2_ Nanotube Array Films with High Photocurrent Density by Spotting Sample Method

**DOI:** 10.1186/s11671-015-1089-7

**Published:** 2015-10-01

**Authors:** Hong Sun, Peini Zhao, Fanjun Zhang, Yuliang Liu, Jingcheng Hao

**Affiliations:** Key Laboratory of Colloid and Interface Chemistry & Key Laboratory of Special Aggregated Materials, Shandong University, Ministry of Education, Jinan, 250100 People’s Republic of China

**Keywords:** Nanotube array films, Photocurrent density, Quantum dots, Spotting sample method

## Abstract

Ag_2_S/CdS/TiO_2_ hybrid nanotube array films (Ag_2_S/CdS/TNTs) were prepared by selectively depositing a narrow-gap semiconductor—Ag_2_S (0.9 eV) quantum dots (QDs)—in the local domain of the CdS/TiO_2_ nanotube array films by spotting sample method (SSM). The improvement of sunlight absorption ability and photocurrent density of titanium dioxide (TiO_2_) nanotube array films (TNTs) which were obtained by anodic oxidation method was realized because of modifying semiconductor QDs. The CdS/TNTs, Ag_2_S/TNTs, and Ag_2_S/CdS/TNTs fabricated by uniformly depositing the QDs into the TNTs via the successive ionic layer adsorption and reaction (SILAR) method were synthesized, respectively. The X-ray powder diffraction (XRD), scanning electron microscopy (SEM), transmission electron microscopy (TEM), and X-ray photoelectron spectrum (XPS) results demonstrated that the Ag_2_S/CdS/TNTs prepared by SSM and other films were successfully prepared. In comparison with the four films of TNTs, CdS/TNTs, Ag_2_S/TNTs, and Ag_2_S/CdS/TNTs by SILAR, the Ag_2_S/CdS/TNTs prepared by SSM showed much better absorption capability and the highest photocurrent density in UV-vis range (320~800 nm). The cycles of local deposition have great influence on their photoelectric properties. The photocurrent density of Ag_2_S/CdS/TNTs by SSM with optimum deposition cycles of 6 was about 37 times that of TNTs without modification, demonstrating their great prospective applications in solar energy utilization fields.

## Background

As useful wide-bandgap semiconductor materials, titanium dioxide (TiO_2_) has been extensively used in wastewater treatment [1], photocatalysis [2–4], gas sensors [5, 6], photochemical water splitting [7, 8], solar cells [9, 10], etc. Because of the broad applications of TiO_2_ and development of nanotechnology, various TiO_2_ structured materials have been synthesized in recent years, such as nanoparticles [11, 12], mesoporous materials [4], nanofilms [13], nanowires [2, 14], nanobelts [15], nanorods [16], and nanotubes [17, 18]. With large surface area and high aspect ratio, nanotubes are nontoxic and environmental friendly, which have attracted much attention in many fields. In 1999, Zwilling et al. reported the preparation of the TiO_2_ nanotube arrays (TNTs) using electrochemical anodization [19]. Since then, many studies have focused on TNTs with advanced structures and improved properties [10, 20–23]. Unlike general TiO_2_ nanotubes with random arrays [18], light comes readily inside TNTs and electrons transfer freely due to the vertical structure and close packing inside the nanotube arrays.

With a bandgap of 3.2 eV, TiO_2_ can absorb only ultraviolet light with a wavelength less than 380 nm, which leads to a very low efficiency of sunlight utilization. In order to solve this problem, the TNTs are usually modified using various methods. Dye sensitization [24] and doping noble metals [25] or semiconductor materials [26] are typical methods in the preparation of hybrid TNT materials, which can widen spectral response range to visible light and exhibit better photoelectric properties. Among various photosensitizers, some large bandgap inorganic semiconductor quantum dots (QDs), such as CdS [26–28], CdSe [29], and PbS [30], are usually used as dopants. These QDs have different valence band and conduction band energy from TiO_2_. In addition to widen the range of visible light response, the QDs/TiO_2_ hybrids can improve charge separation capability and minimize charge carrier recombination probabilities. However, little has been focused on the use of small bandgap semiconductors in the field of sensitizers because photoelectrons and holes recombine readily, though they may absorb a wide range of sunlight.

To our best knowledge, nearly all sensitizers are uniformly distributed on TiO_2_. In this study, we prepared the hybrid nanotube array films of Ag_2_S/CdS/TNTs by spotting sample method (SSM). Firstly, nanoporous TNTs were prepared using electrochemical anodization under controlled reaction conditions, which have better photoelectric properties [23]; secondly, CdS was deposited inside TNTs via the successive ionic layer adsorption and reaction (SILAR) method, which can widen visible light response spectrum; thirdly, Ag_2_S was deposited in the local domain of the CdS/TNTs using spotting sample method. As a narrow-bandgap (0.9 eV) semiconductor material, Ag_2_S strongly absorbs visible light. However, too much Ag_2_S deposition can lead to clog-up of nanotube inlet, which could decrease the sunlight absorption capabilities of TiO_2_ nanotubes. In order to overcome this problem, a narrow-gap semiconductor material, Ag_2_S, was deposited in the local domain of CdS/TNTs by spotting sample method, which may result in a lower coverage of Ag_2_S on TNTs, and the presence of these three semiconductor materials can decrease the recombination possibilities of photoelectrons and holes. Five different films, Ag_2_S/CdS/TNTs by SSM, TNTs, CdS/TNTs, Ag_2_S/TNTs, and Ag_2_S/CdS/TNTs by SILAR, were prepared and analyzed to compare their photoelectro-chemical properties.

## Methods

### Chemicals and Materials

All the chemicals used are analytical grade, and the titanium foil (0.3 mm thick, purity > 99.9 %) is obtained from Sumitomo. Milli-Q water is from a three-stage Milli-Q plus 185 purification system with a resistivity larger than 18.2 MΩ · cm.

### Preparation of TiO_2_ Nanotube Array Films

TNTs were prepared by anodic oxidation. Titanium foils were tailored into small pieces (2.5 × 3.0 mm). In order to be dust-free and oil-free on the titanium foils, titanium pieces were ultrasonic cleaned for 30 min in deionized water and acetone, respectively. After that, titanium foils were polished in mixed solution of HF (40 %):HNO_3_ (65 %):H_2_O = 1:4:5 (volume) for 3 min, then were rinsed in Milli-Q water and dried in nitrogen gas. Finally, anodic oxidation was carried out in electrolyte solution (0.8 g NH_4_F + 150 g HOCH_2_CH_2_OH + 0.5 mL 40 % HF) at 10 °C for 30 min. The crystalline phase anatase-TiO_2_ was obtained using thermal treatment at 450 °C for a period of 2 h. In this experiment, the constant voltage was 60 V, current is 0.07~0.10 A/cm^2^, the titanium foils were used as anode, and a graphite rod served as a counter electrode.

### Preparation of Ag_2_S/CdS/TiO_2_ Hybrid Nanotube Array Films

**Fig. 1:**
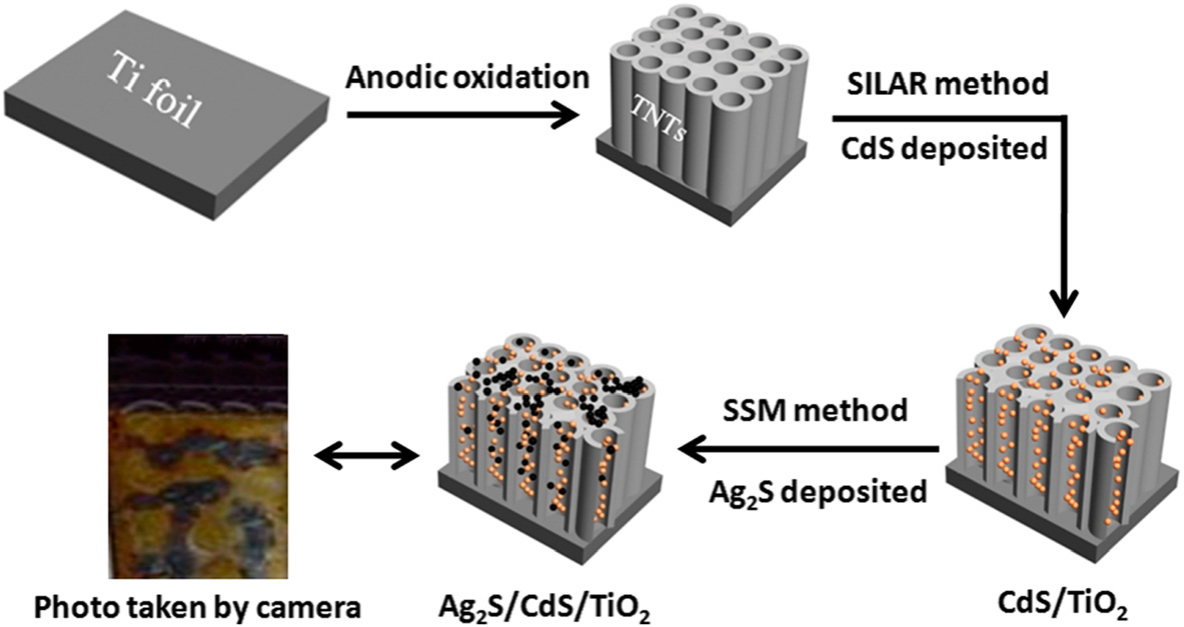
The synthesis process of Ag_2_S/CdS/TNTs by SSM

CdS/TNTs were fabricated via the successive ionic layer adsorption and reaction (SILAR) method. The TNTs were dipped inside 0.025 mol · L^−1^ Cd(NO_3_)_2_ and 0.025 mol · L^−1^ Na_2_S aqueous solutions for 5 min separately and for a couple of cycles. Before dipping into each solution, the nanotube array films were simply rinsed with Milli-Q water and dried in N_2_ stream. This treatment procedure was repeated depending on our need. Ag_2_S/TNTs were fabricated via similar methods. After being washed in Milli-Q water and dried in N_2_ stream, CdS/TNTs were ready for the Ag_2_S/CdS/TNTs preparation via SSM. A 0.025 mol · L^−1^ AgNO_3_ solution (300 μL) was spotted on the local domain of CdS/TNTs with a capillary glass tube (0.5 mm) and dried in air; then, a 0.025 mol · L^−1^ Na_2_S solution (150 μL) was spotted on the same place and dried in air. The preparation procedure was repeated a number of times according to requirement. After being washed in ultrapure water, the hybrid nanotube array films were thermally treated at 200 °C for 2 h. The solution used in this work is ethanol/water solution (ethanol:water = 1:1). The synthesis process of Ag_2_S/CdS/TNTs by SSM is shown in Fig. 1. Correspondingly, Ag_2_S/CdS/TNTs was fabricated by uniformly depositing the Ag_2_S quantum dots into the CdS/TNTs via SILAR method.

### Characterizations

Surface morphology of nanotube array films and direct cross section of TNTs thickness measurements were carried out using a JEOL JSM-6700F field emission scanning electron microscopy (FE-SEM). The morphology and microstructure of TNTs coupled with CdS and Ag_2_S.

QDs were characterized using a JEOL JEM-1400 transmission electron microscope (TEM) and a JEOL JEM-2100F high-resolution transmission electron microscope (HR-TEM). Energy dispersive X-ray spectroscopy (EDX) was also carried out in the TEM. The X-ray powder diffraction (XRD) patterns were collected on a Bruker D8 advance X-ray diffractometer with Cu Kα (*λ* = 0.15418 nm) radiation for structure analysis. The surface composition of nanotube array films and binding energy were characterized using ESCALAB 250 X-ray photoelectron spectrum (XPS). The optical absorption of TNTs and hybrid QDs/TNTs was characterized using TU-1901 UV-vis diffuse reflectance absorption spectra (DRS).

### Photocurrent Measurements

Photocurrent measurements were carried out with a model 263A potentiostat/galvanostat and a three-electrode test cell without applying any bias. The TNTs, CdS/TNTs, Ag_2_S/TNTs, Ag_2_S/CdS/TNTs by SSM, and Ag_2_S/CdS/TNTs by SILAR method were subsequently used as the working electrode. A platinum wire was used as the counter electrode, and a saturated calomel electrode (SCE) was used as the reference electrode. The measurements were carried out with a 300-W xenon lamp (PLS-SXE300/300UV) as light irradiation source. 0.20 mol · L^−1^ Na_2_S and 0.20 mol · L^−1^ Na_2_SO_3_ aqueous solutions were used as electrolyte with sacrificial agent. The incident photon to current conversion efficiency (IPCE) measurements was performed employing a 150-W Xe lamp coupled with a computer-controlled monochromator. Electrochemical impedance spectroscopy (EIS) was performed under illumination with an AC amplitude of 5 mV and frequency range between 100 kHz and 0.1 Hz.

## Results and Discussion

**Fig. 2:**
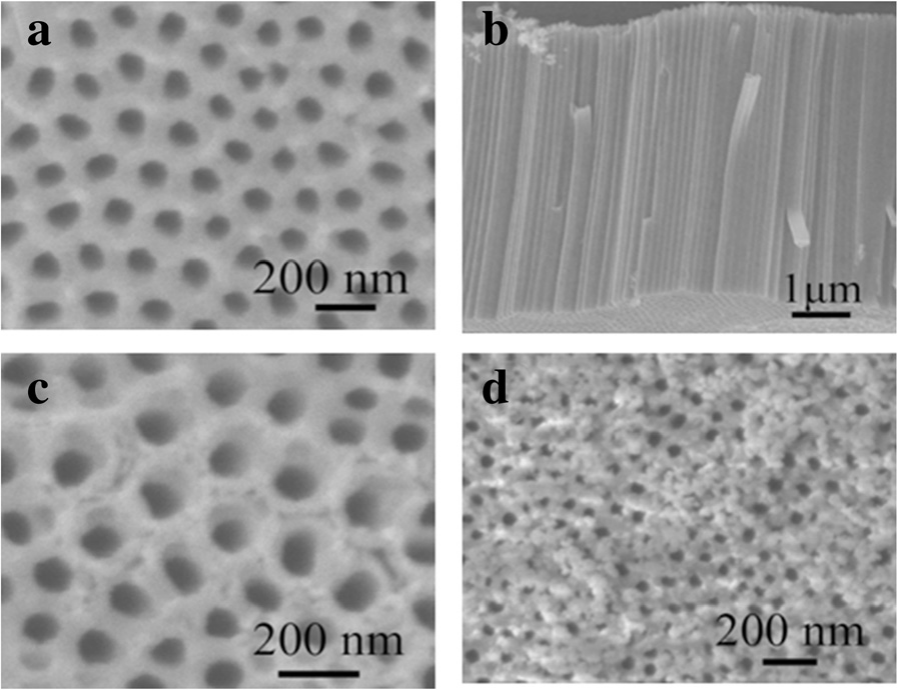
**a**, **b** The SEM images of the top surface morphologies and a view of cracked TiO_2_ nanotubes anodized (10 °C) at 60 V for 30 min. **c** The SEM images of the top surface morphologies of CdS/TNTs fabricated using successive ionic layer adsorption and reaction (SILAR) method. **d** The TEM images of the top surface morphologies of the Ag_2_S/CdS/TNTs obtained via SSM

Figure 2a, b shows the SEM images of the top surface morphologies and a view of cracked TiO_2_ nanotubes anodized (10 °C) at 60 V for 30 min. It is very clear that the shape of the tubes is quite regular and uniform. The inner diameter of the tubes is about 75 nm, and the wall thickness of the TiO_2_ nanotubes on top is ~25 nm. As shown in Fig. 2b, TiO_2_ nanotubes pack closely with each other and the TNT length is about 5.5 μm. The SEM images of the top surface morphologies of CdS/TNTs fabricated using successive ionic layer adsorption and reaction (SILAR) method are shown in Fig. 2c. No particle aggregates can be observed at the inlet of the nanotubes, and the surface roughness of the array films rises dramatically. It may be considered that some CdS nanoparticles gather around the tubes. Figure 2d shows the TEM images of the top surface morphologies of the Ag_2_S/CdS/TNTs obtained via SSM. As shown in the images, a small part of the tubes is blocked by the Ag_2_S particles, which depends on the deposition cycles of Ag_2_S, and sunlight can still enter the unblocked tubes.

**Fig. 3:**
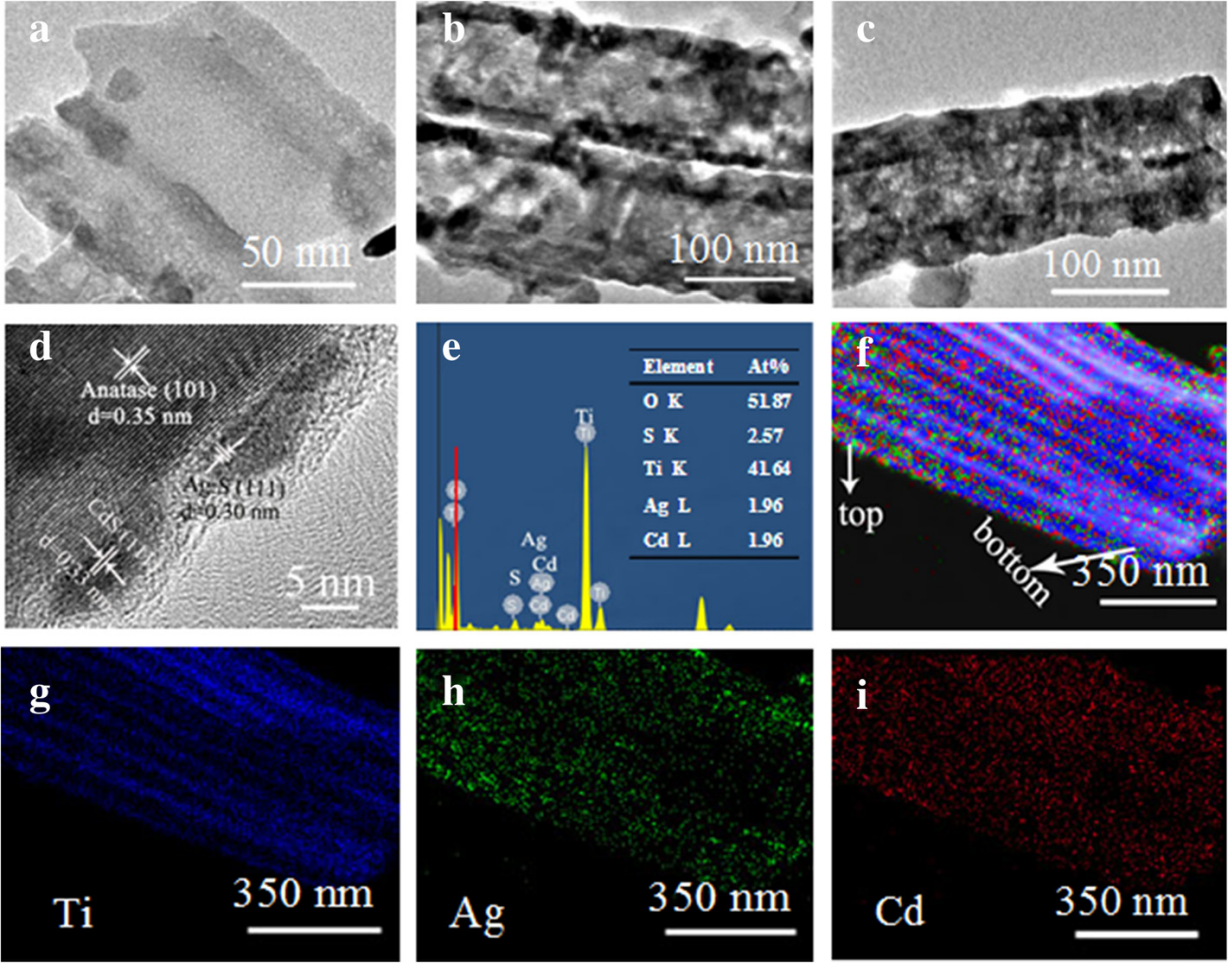
**a**–**i** The TEM and HR-TEM images of TNTs (**a**), CdS/TNTs (**b**), and Ag_2_S/CdS/TNTs by SSM (**c,d**). EDX spectrum (**e**) of the Ag_2_S/CdS/TNTs by SSM (the inset is the table of elements content) and the corresponding EDX elemental mapping (**f**) of the total elemental mapping of major elements (Ti, Cd and Ag).

Figure 3 shows the TEM and HR-TEM images of TNTs, CdS/TNTs, and Ag_2_S/CdS/TNTs by SSM. As seen from the TEM images of fracture surfaces of a TiO_2_ nanotube (Fig. 3a), the inner surface is very smooth. Figure 3b shows that CdS particles get into the TiO_2_ nanotubes. Unlike Fig. 3a, some particles were introduced inside the nanotubes. For porous TNTs, the tubes pack closely with each other and CdS did not deposit on the outer wall of the TiO_2_ nanotubes. Figure 3c shows the TEM image of the TiO_2_ nanotubes deposited with CdS and Ag_2_S. From the TEM images, one can see that there is no obvious difference between Fig. 3c and Fig. 3b HR-TEM images; Fig. 3d shows some particles deposited on the wall of the TiO_2_ nanotube. Lattice fringes of 0.35 nm were observed in detailed microscopic structures of the TiO_2_ nanotubes, corresponding to the (101) plane of anatase (JCPDS file no. 71-1167), suggesting that the TiO_2_ nanotubes are well crystalline structures. The particle diameters are about 6~10 nm inside the TiO_2_ nanotubes, and the lattice fringes are obviously different from the lattice fringes of the TiO_2_ nanotubes. These particles are CdS or Ag_2_S. Lattice fringes of 0.30 nm were observed, which corresponds to the (111) plane of the acanthite Ag_2_S (JCPDS file no. 14-0072), and approximately 0.33 nm corresponds to the (111) plane of the cubic phase of CdS (JCPDS file no. 80-0019). In order to further identify the elemental composition and where CdS and Ag_2_S particles were deposited, an area of TNTs deposited with CdS and Ag_2_S is chosen to analyze the corresponding EDX and EDX elemental mapping. As shown in Fig. 3e, O, S, Ti, Ag, and Cd can be identified. The quantitative analysis reveals that the atomic composition of O, S, Ti, Ag, and Cd is 51.87, 2.57, 41.64, 1.96, and 1.96 %, respectively. With a molar ratio of Ag to Cd at 1:1, we may calculate the molar ratio of Ag_2_S to CdS at 1:2. We calculated that S should be 2.94 %, which is close to 2.57 % and confirms the formation of Ag_2_S and CdS. Elemental mapping of Ag_2_S/CdS/TNTs-S, Fig. 3f–i, shows that Ag_2_S and CdS were deposited inside the TiO_2_ nanotubes uniformly.

**Fig. 4:**
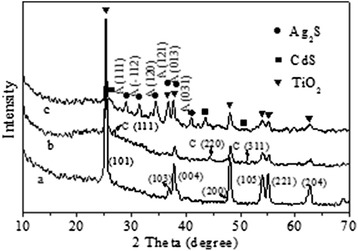
The composition and crystalline structures of the TNTs (**a**), CdS/TNTs (**b**), and Ag_2_S/CdS/TNTs (**c**) by SSM characterized using XRD and the XRD patterns (A for Ag_2_S, and C for CdS; solid circle for Ag_2_S, solid square for CdS, and solid triangle for TiO_2_)

The composition and crystalline structures of the TNTs, CdS/TNTs, and Ag_2_S/CdS/TNTs by SSM are also characterized using XRD, and the XRD patterns are shown in Fig. 4. Figure 4 (a) shows the characteristic diffraction peaks of anatase TiO_2_ (JCPDS file no. 71-1167). The diffraction peaks confirm that TiO_2_ nanotubes are anatase phase after thermal treatment at 450 °C for 2 h. The new weak peaks except the peaks of anatase TiO_2_ appearing in Fig. 4 (b) are CdS peaks (JCPD file no. 80-0019, marked by C), and these peaks reveal that the CdS particles have the cubic structure. As shown in Fig. 4 (c), all the diffraction peaks of Ag_2_S, CdS, and TiO_2_ are marked with solid circle, solid square, and solid triangle. The peaks of Ag_2_S attribute to acanthite (JCPDS file no. 14-0072, marked by A). The XRD patterns further confirm the HR-TEM, EDX, and elemental mapping results.

**Fig. 5:**
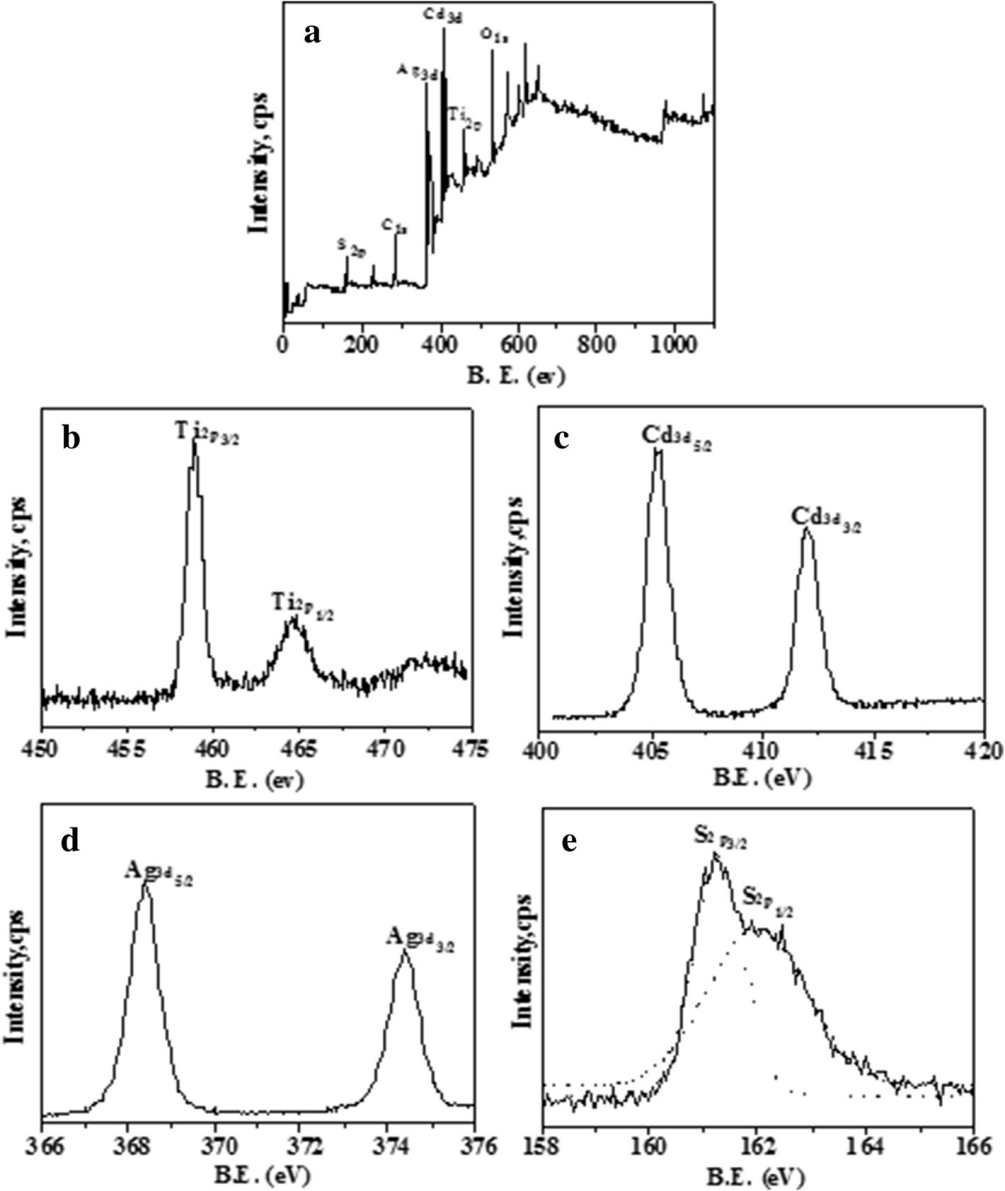
**a** The S, C, Ag, Cd, Ti, and O elements can be found in the survey spectrum. **b** The high-resolution Ti2p XPS spectrum has two peaks at 464.8 and 458.9 eV. **c** Two characteristic peaks are observed at 405.2 (Cd3d_5/2_)  and 412.0 eV (Cd3d_3/2_). **d** The Ag3d core level could be satisfactorily fit to single spin-orbit pair at 368.4 eV (Ag3d_5/2_) and at 374.4 eV (Ag3d_3/2_). **e** The high-resolution XPS spectrum of S2p

The X-ray photoelectron spectroscopy (XPS) was carried out to further confirm the chemical state of Ti, S, Ag, and Cd atoms in the array films of Ag_2_S/CdS/TNTs by SSM, and each element gives rise to a characteristic set of peaks. The S, C, Ag, Cd, Ti, and O elements can be found in the survey spectrum (Fig. 5a). The C element is mainly from carbon grid. The high-resolution Ti2p XPS spectrum (Fig. 5b) has two peaks at 464.8 and 458.9 eV, and the doublet feature due to spin-orbit splitting results in the 2p_3/2_ and 2p_1/2_ peaks with spin-orbit separation 5.9 eV. This result agrees with Ti(IV) in pure anatase TiO_2_. As shown in Fig. 5c, two characteristic peaks are observed at 405.2 and 412.0 eV, which belong to Cd3d_5/2_ and Cd3d_3/2_, respectively. The Ag3d core level could be satisfactorily fit to single spin-orbit pair at 368.4 eV (Ag3d_5/2_) and at 374.4 eV (Ag3d_3/2_) in Fig. 5d. Figure 5e is the high-resolution XPS spectrum of S2p, and two peaks have also been given through Gaussian fitting. The two characteristic peaks at 161.2 and 162.2 eV are assigned to S2p_3/2_ and S2p_1/2_. With reference to the literature [31], the peak S2p_1/2_ at 162.2 eV corresponds to CdS and the peak S2p_3/2_ at 161.2 eV to Ag_2_S.

**Fig. 6:**
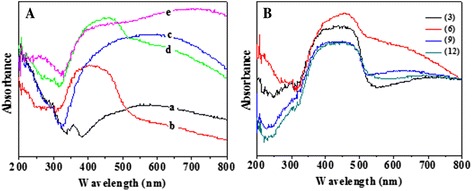
**A** The UV-vis absorption spectra of array films of TNTs (a), CdS/TNTs (b), Ag_2_S/TNTs (c), Ag_2_S/CdS/TNTs by SSM (d), and Ag_2_S/CdS/TNTs via SILAR (e). **B** The UV-vis absorbance of the Ag_2_S/CdS/TNTs by SSM with different deposition cycles of CdS and TiO_2_

Figure 6a shows the UV-vis absorption spectra of array films of TNTs, CdS/TNTs, Ag_2_S/TNTs, Ag_2_S/CdS/TNTs by SSM, and Ag_2_S/CdS/TNTs via SILAR. The Ag_2_S/CdS/TNTs via SILAR was produced by depositing Ag_2_S via SILAR method on the whole film of CdS/TNTs. Both CdS and Ag_2_S were deposited for 6 cycles. The addition of CdS resulted in an additional absorption band in the UV-vis region (320–530 nm). Compared with TNTs, the absorption of Ag_2_S/TNTs extends to a longer wavelength up to 800 nm, corresponding to the narrow bandgap of Ag_2_S (0.9 eV). Both Ag_2_S/CdS/TNTs by SSM and Ag_2_S/CdS/TNTs via SILAR show an obvious absorption in light range (320–800 nm), indicating that the Ag_2_S and CdS deposited onto the TiO_2_ nanotube arrays can increase the visible light absorption. As shown in Fig. 6a (a), TNTs fabricated by anodic oxidation not only have strong absorption in the UV region but also have a certain degree of absorption in visible range (400–800 nm). When CdS particles were deposited on the TNTs, the absorption of the TNTs was decreased accordingly in visible range (400–800 nm). So, as shown in Fig. 6a (b), CdS/TNTs has a lower absorbance than TNTs under the visible spectrum (500–800 nm). This is also the reason that sample d (Ag_2_S/CdS/TNTs by SSM) has a lower absorbance than sample c (Ag_2_S/TNTs) under the visible light spectrum (500–800 nm). Figure 6b shows the UV-vis absorbance of the Ag_2_S/CdS/TNTs by SSM with different deposition cycles of CdS and TiO_2_, and the absorbance increases firstly and then decreases with the increase of the deposition cycles. The Ag_2_S/CdS/TNTs by SSM with 6 deposition cycles has stronger absorbance in UV-vis range, which indicates that an optimum amount of doping of Ag_2_S and CdS can benefit the optical property of TNTs. At first, the absorption of Ag_2_S/CdS/TNTs by SSM under UV-vis regions increased with an increase of Ag_2_S and CdS particles. But, with further depositing and aggregation of the particles, large particles exhibit increased light scattering [32], so the absorbance of Ag_2_S/CdS/TNTs by SSM then decreased.

**Fig. 7:**
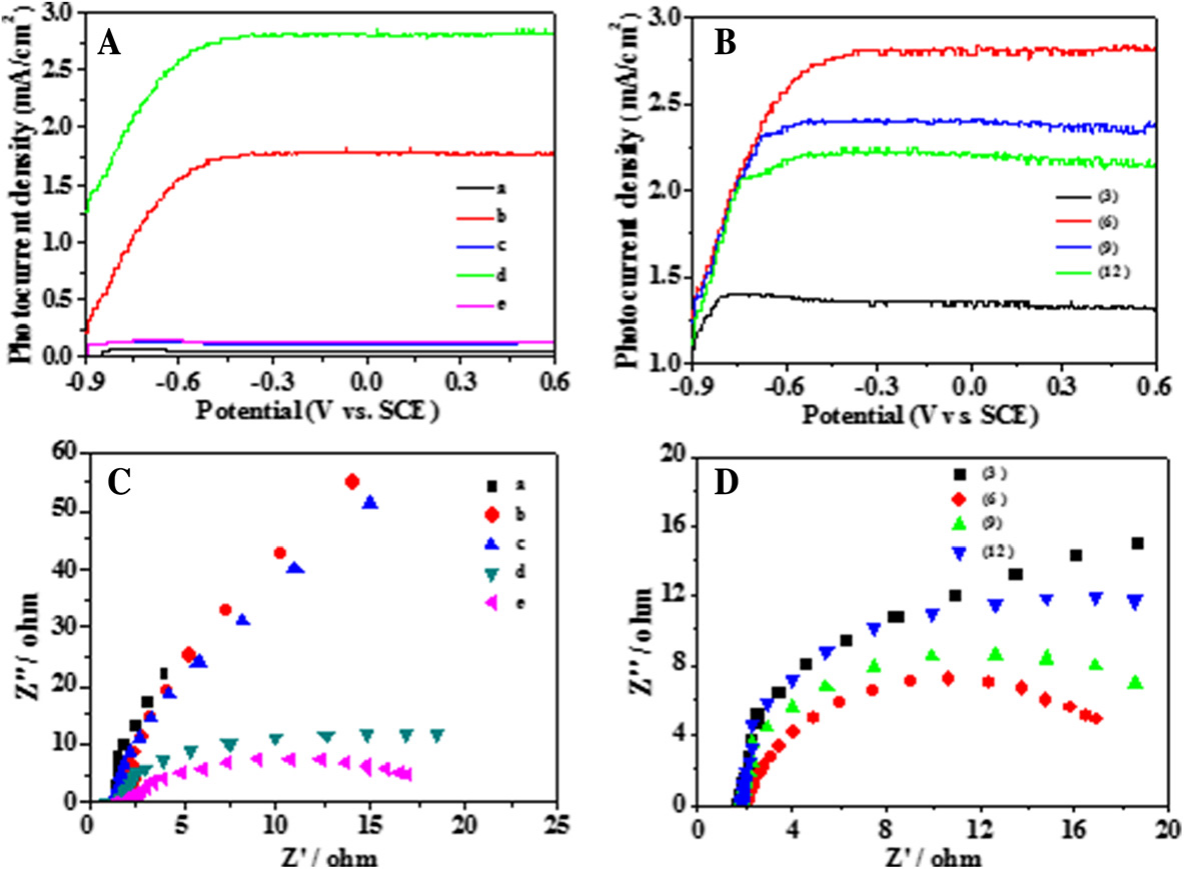
**A**, **B** The photocurrent density (*I*-*V*) curves of different array films to evaluate their photoelectric properties. **C**, **D** The EIS Nyquist plots. TNTs (a), CdS/TNTs (b), Ag_2_S/TNTs (c), Ag_2_S/CdS/TNTs by SSM (d), and Ag_2_S/CdS/TNTs via SILAR (e). All the QDs are deposited 6 cycles (**A** and **C**), Ag_2_S/CdS/TNTs by SSM at different deposition cycles (**B** and **D**), the number in the parenthesis is the deposition cycles. The experiments were carried out under irradiation of visible light (λ > 420 nm).

Figure 7a, b shows the photocurrent density (*I*-*V*) curves of different array films to evaluate their photoelectric properties. As shown in Fig. 7a, under visible light illumination, TNTs, Ag_2_S/TNTs, and Ag_2_S/CdS/TNTs via SILAR exhibit low photocurrent density, while high photocurrent density values were recorded for CdS/TNTs and Ag_2_S/CdS/TNTs by SSM. Clearly, Ag_2_S/CdS/TNTs by SSM exhibits the highest photocurrent density among the five array films of TNTs, CdS/TNTs, Ag_2_S/TNTs, Ag_2_S/CdS/TNTs by SSM, and Ag_2_S/CdS/TNTs via SILAR method. The photocurrent density of Ag_2_S/CdS/TNTs by SSM (2.83 mA/cm^2^ at 0 V vs. SCE) is much higher than that of TiO_2_ array films (0.59 mA/cm^2^). In addition, the onset potentials of all the array films are read directly from the instrument. The onset potentials of TiO_2_, CdS/TiO_2_, Ag_2_S/TiO_2_, Ag_2_S/CdS/TNTs via SILAR, and Ag_2_S/CdS/TNTs by SSM are −0.85, −1.09, −0.89, −0.90, and −1.21 V, respectively. The negative shift in onset potential values indicates the decreased surface state densities of the electrodes and increased charge transfer rates at the interface [33], suggesting that Ag_2_S/CdS/TNTs by SSM has improved photoelectric properties compared with other array films. As shown in Fig. 7b, Ag_2_S/CdS/TNTs by SSM with 6 deposition cycles exhibits the largest photocurrent density, and the onset potentials of Ag_2_S/CdS/ TNTs by SSM with 3, 6, 9, and 12 deposition cycles are −0.96, −1.21, −1.09, and −1.05 V, respectively. In order to investigate the photoelectric properties of the five nanotube array films further, electrochemical impedance spectra (EIS) measurements were carried out using the five films respectively as the working electrodes in 0.20 M Na_2_S/Na_2_SO_3_ aqueous solution at the open-circuit potential of the system under visible illumination (*λ* > 420 nm) with an AC amplitude of 5 mV and frequency range from 100 kHz to 0.1 Hz. The EIS Nyquist plots were shown in Fig. 7c, d. As depicted in Fig. 7c, the diameters of the impedance arcs of CdS/TNTs and Ag_2_S/CdS/TNTs by SSM are smaller than those of TNTs, Ag_2_S/TNTs, and Ag_2_S/CdS/TNTs via SILAR. In the EIS Nyquist plots, the smaller semicircle diameter indicates an effective separation of photogenerated electron-hole pairs and fast interfacial charge transfer to the electron donor or acceptor. Obviously, the impedance arc of Ag_2_S/CdS/TNTs by SSM is the smallest among the five films of TNTs, CdS/TNTs, Ag_2_S/TNTs, Ag_2_S/CdS/TNTs via SILAR, and Ag_2_S/CdS/TNTs by SSM, confirming that the synergy of Ag_2_S, CdS, and TiO_2_ may decrease the interfacial resistance and increase the separation capability of photogenerated electron-hole pairs of Ag_2_S/CdS/TNTs by SSM. However, Ag_2_S/TNTs and Ag_2_S/CdS/TNTs via SILAR have larger semicircle diameters despite a higher absorption capability of visible light. This suggests that uniformly deposited Ag_2_S on the whole TNTs may increase the recombination capability of photogenerated electron-hole pairs and block the electron transfer to Ti substrate. In addition, as shown in Fig. 7d, the deposition cycles of CdS and Ag_2_S affect the impedance arc of Ag_2_S/CdS/TNTs by SSM. Ag_2_S/CdS/TNTs by SSM with 6 deposition cycles has the smallest semicircle diameter, which suggests that an optimal number of deposition cycles may improve its photoelectric properties obviously. Too much QD deposition will increase interfacial resistance and the recombination possibility of the photogenerated electron-hole pairs. The EIS results of the different array films correspond to the changing trends of photocurrents and the onset potentials (Fig. 7a, b).

**Fig. 8:**
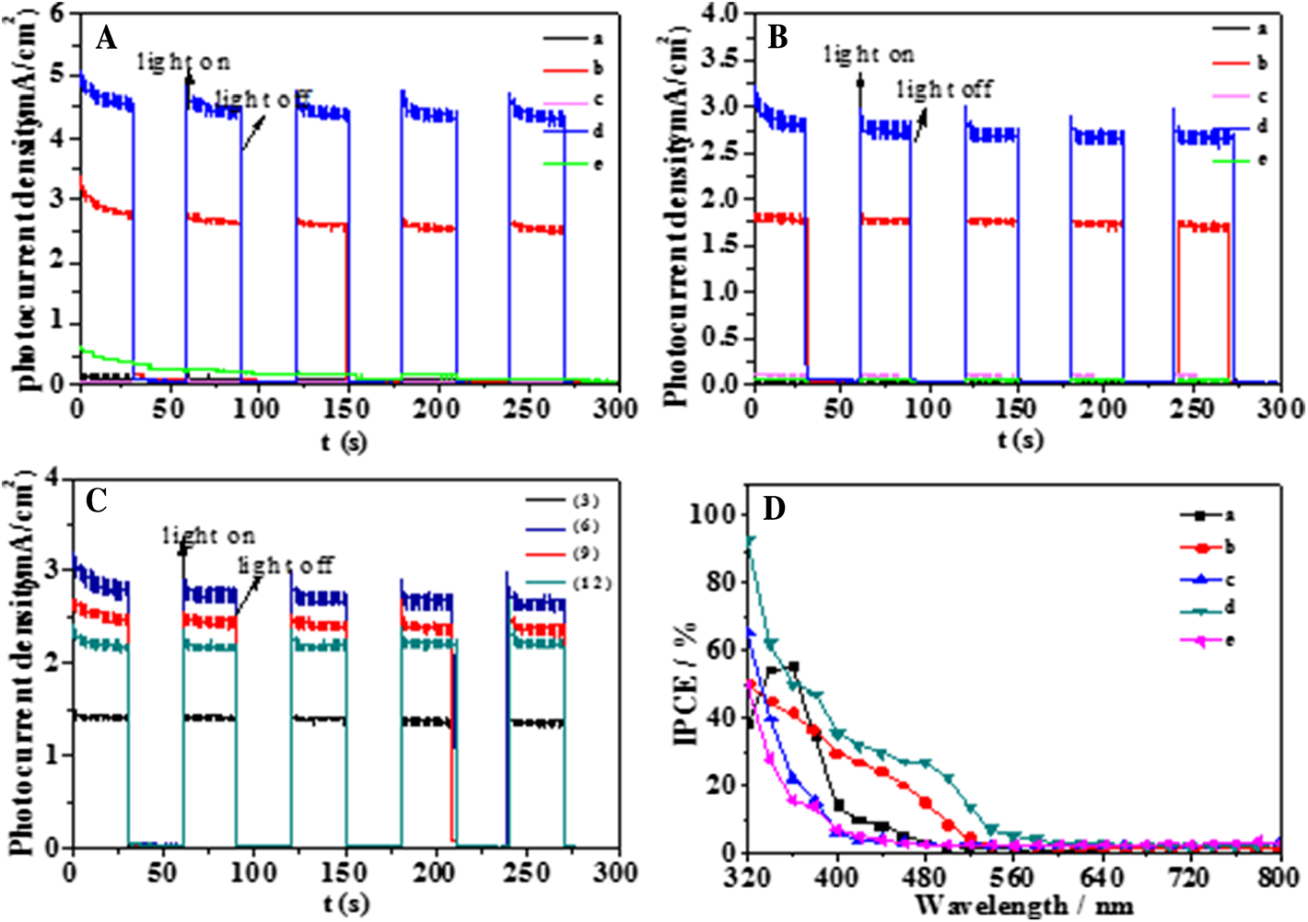
**A** The *I*-*t* curves of the five films under intermittent illumination of UV and visible light (320–800 nm) at bias voltages of 0 V vs. reference electrode Hg/Hg_2_Cl_2_. **B** The *I*-*t* curves of the five different anodes under intermittent illumination of visible light (*λ* > 420 nm). **C** The photocurrent response of the array films of Ag_2_S/CdS/TNTs by SSM with different deposition cycles. **D** The photocurrent-response of pure TNTs to the incident light with various wavelengths is principally active in the UV light region. TNTs (a), CdS/TNTs (b), Ag_2_S/TNTs (c), Ag_2_S/CdS/TNTs by SSM (d), and Ag_2_S/CdS/TNTs via SILAR (e)

Figure 8a shows the *I*-*t* curves of the five films under intermittent illumination of UV and visible light (320–800 nm) at bias voltages of 0 V vs. reference electrode Hg/Hg_2_Cl_2_. TNTs, CdS/TNTs, Ag_2_S/TiO_2_, and Ag_2_S/CdS/TNTs by SSM show an instantaneous change in current upon illumination and the current responses in the dark are negligible, indicating that the separation of the electrons and holes of the above four array films is prompt and the charge transport inside the films is fast. The photocurrent density of Ag_2_S/CdS/TNTs by SSM (average 4.5 mA/cm^2^) is higher than that of CdS/TNTs (average 3.0 mA/cm^2^). Unlike CdS/TNTs and Ag_2_S/CdS/TNTs by SSM, the TNTs, Ag_2_S/TNTs, and Ag_2_S/CdS/TNTs via SILAR show very low photocurrent density values under Xe light irradiation. Photocurrent density of hybrid Ag_2_S/CdS/TNTs by SSM is about 37 times higher than that of pure TNTs (average 0.14 mA/cm^2^). Interestingly, the photocurrent of the array films of Ag_2_S/CdS/TNTs via SILAR is very small and decreases gradually, demonstrating that the photoelectrons and holes may recombine rapidly. These results indicate that more free carriers are generated and quickly transfer in Ag_2_S/CdS/TNTs by SSM than in other array films. Figure 8b shows the *I*-*t* curves of the five different anodes under intermittent illumination of visible light (*λ* > 420 nm). Similar to Fig. 8a, the photocurrent density of Ag_2_S/CdS/TNTs by SSM (average 2.8 mA/cm^2^) and CdS/TNTs (average 1.8 mA/cm^2^) is much higher than that of TNTs, Ag_2_S/TNTs, and Ag_2_S/CdS/TNTs via SILAR, which indicates lower visible light response of pure TNTs. Figure 8c provides the photocurrent response of the array films of Ag_2_S/CdS/TNTs by SSM with different deposition cycles. Ag_2_S/CdS/TNTs by SSM with 6 deposition cycles generates the largest photocurrent than those of the samples with different deposition cycles. The incident photon to current efficiency (IPCE) test of the five different films (TNTs, CdS/TNTs, Ag_2_S/TNTs, Ag_2_S/CdS/TNTs by SSM, and Ag_2_S/CdS/TNTs via SILAR) was carried out at 0 V vs. Hg/Hg_2_Cl_2_. As shown in Fig. 8d, the photo-response of pure TNTs to the incident light with various wavelengths is principally active in the UV light region. Compared with TNTs, CdS/TNTs and Ag_2_S/CdS/TNTs by SSM exhibit higher IPCE value, and Ag_2_S/CdS/ TNTs by SSM shows the highest photocurrent conversion efficiency at a wavelength range between 320 and 600 nm. However, the IPCE value of Ag_2_S/TNTs and Ag_2_S/CdS/TNTs via SILAR is lower than that of TNTs at a wavelength from 330 to 500 nm.

**Fig. 9:**
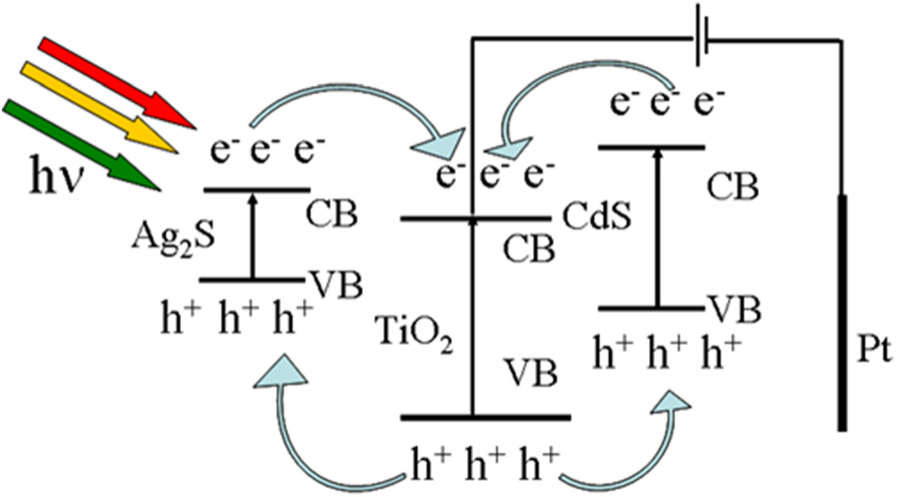
The schematic photovoltaic conversion of Ag_2_S/CdS/TNTs by SSM

In reference to the mechanism proposed in literatures [34–37], the schematic photovoltaic conversion of Ag_2_S/CdS/TNTs by SSM is shown in Fig. 9. The photogenerated electrons separate from the photogenerated holes under light irradiation. However, the photogenerated electrons and holes recombine with each other if the electrons cannot be transferred. The conduction band (CB) of the semiconductor (CdS) is located above the CB level of the TNTs, and the photogenerated electrons can transfer from the CB of the CdS to the CB of the adjacent TNTs. The electrons flow away through Ti substrate and wires rapidly. The valence band (VB) of the TNTs is below that of the adjacent CdS, the photogenerated holes transfer from the VB of the TNTs to that of the CdS, and the holes transfer to the surface of Ag_2_S/CdS/TNTs by SSM. The two factors can reduce the recombination probability of electrons and holes. The similar transfer process of the photogenerated electrons and holes of Ag_2_S and TNTS can happen. With a lower bandgap energy (0.9 eV), Ag_2_S has a wider range of light absorption, and photogenerated electrons and holes are easier to separate than wide-bandgap semiconductors, though they are ready to recombine at the same time, which can be reduced by the interaction between Ag_2_S, CdS and TiO_2_. However, the photogenerated electron numbers of Ag_2_S/CdS/TNTs via SILAR can be reduced because such a large amount of Ag_2_S uniformly deposited on the whole CdS/TNTs may reduce the light absorption of CdS and TNTs. Under irradiation, the photogenerated holes transferred to Ag_2_S and CdS, which will reduce the stability of Ag_2_S/CdS/TNTs by SSM in practical applications. So, 0.20 M Na_2_S and 0.20 M Na_2_SO_3_ aqueous solutions were used as electrolyte with sacrificial agent, and the holes in the Ag_2_S and CdS with active oxidation ability were captured by S^2−^ and S_2_O_3_^2−^ in the electrolyte (2h^+^ + S^2 −^ + SO_3_^2 −^ → S_2_O_3_^2 −^) [38], which can enhance the stability of the Ag_2_S/CdS/TNTs by SSM.

## Conclusions

Ag_2_S/CdS/TNTs by SSM are fabricated via spotting sample method. SEM, TEM, XRD, and XPS results show that most of the CdS and Ag_2_S can be deposited into the TNTs and some Ag_2_S particles are deposited on the surface of the array films. The Ag_2_S nanoparticles deposited in the local range of the CdS/TNTs and Ag_2_S/CdS/TNTs by SSM may widen the absorption spectra of TNTs significantly to the visible light region and even enhance the absorption of the UV light (320–800 nm). Unlike TNTs, CdS/TNTs, Ag_2_S/TNTs, and Ag_2_S/CdS/TNTs via SILAR, Ag_2_S/CdS/TNTs by SSM have a much larger photocurrent under UV-vis light irradiation. The photocurrent density of Ag_2_S/CdS/TNTs by SSM with an optimal number of deposition cycles of 6 was about 37 times than that of TNTs under light (320–800 nm) irradiation of Xe lamp. With better photoelectric properties and stability of Ag_2_S/CdS/TNTs by SSM, they have good prospective application in solar energy utilization fields.
